# Heterogeneity of glycaemic phenotypes in type 1 diabetes

**DOI:** 10.1007/s00125-024-06179-4

**Published:** 2024-05-23

**Authors:** Guy Fagherazzi, Gloria A. Aguayo, Lu Zhang, Hélène Hanaire, Sylvie Picard, Laura Sablone, Bruno Vergès, Naïma Hamamouche, Bruno Detournay, Michael Joubert, Brigitte Delemer, Isabelle Guilhem, Anne Vambergue, Pierre Gourdy, Samy Hadjadj, Fritz-Line Velayoudom, Bruno Guerci, Etienne Larger, Nathalie Jeandidier, Jean-François Gautier, Eric Renard, Louis Potier, Pierre-Yves Benhamou, Agnès Sola, Lyse Bordier, Elise Bismuth, Gaëtan Prévost, Laurence Kessler, Emmanuel Cosson, Jean-Pierre Riveline, on behalf of the SFDT1 study team

**Affiliations:** 1 Deep Digital Phenotyping Research Unit, Department of Precision Health, Luxembourg Institute of Health, Strassen, Luxembourg; 2 Bioinformatics Platform, Luxembourg Institute of Health, Strassen, Luxembourg; 3 Department of Diabetology, Metabolic Diseases and Nutrition, CHU Toulouse, University of Toulouse, Toulouse, France; 4 Francophone Foundation for Diabetes Research, Paris, France; 5 Endocrinology and Diabetes, Point Medical, Dijon, France; 6 Department of Endocrinology-Diabetology, Inserm LNC UMR1231, University of Burgundy, Dijon, France; 7 e-Health Services Sanoïa, Gémenos, France; 8 CEMKA, Bourg-la-Reine, France; 9 Service d’Endocrinologie-Diabétologie (Endocrinology/Diabetes Unit), Centre Hospitalier Universitaire de Caen, Caen, France; 10 Endocrinology, Diabetology and Nutrition Department, Robert Debré University Hospital, Reims, France; 11 Department of Endocrinology, Diabetes and Nutrition, University Hospital of Rennes, Rennes, France; 12 Endocrinology, Diabetology, Metabolism and Nutrition Department, Lille University Hospital, Lille, France; 13 Institute of Metabolic and Cardiovascular Diseases, UMR1297 Inserm/UPS, Toulouse University, Toulouse, France; 14 Institut du thorax, INSERM, CNRS, Université Nantes, CHU Nantes, Nantes, France; 15 Department of Endocrinology-Diabetology, University Hospital of Guadeloupe, Pointe-À-Pitre, France; 16 Inserm UMR1283, CNRS UMR8199, European Genomic Institute for Diabetes (EGID), Lille, France; 17 Department of Endocrinology, Diabetology, and Nutrition, Brabois Adult Hospital, University of Lorraine, Vandoeuvre-Lès-Nancy, France; 18 University Paris Cité, Institut Cochin, U1016, Inserm, Paris, France; 19 Diabetology Department, Cochin Hospital, AP-HP, Paris, France; 20 Department of Endocrinology, Diabetes and Nutrition, Hôpitaux Universitaires de Strasbourg, Université de Strasbourg, Strasbourg, France; 21 Institut Necker Enfants Malades, Inserm U1151, CNRS UMR 8253, IMMEDIAB Laboratory, Paris, France; 22 Centre Universitaire de Diabétologie et de ses Complications, AP-HP, Hôpital Lariboisière, Paris, France; 23 Institute of Functional Genomics, University of Montpellier, CNRS, Inserm, Montpellier, France; 24 Department of Endocrinology, Diabetes, Nutrition, Montpellier University Hospital, Montpellier, France; 25 Department of Diabetology, Endocrinology and Nutrition, AP-HP, Bichat Hospital, Paris, France; 26 Université Grenoble Alpes, Inserm U1055, CHU Grenoble Alpes, Grenoble, France; 27 Service d’Endocrinologie, Hôpital Bégin, Saint Mandé, France; 28 Robert-Debré University Hospital, Department of Paediatric Endocrinology and Diabetology, AP-HP, University of Paris, Paris, France; 29 Department of Endocrinology, Diabetes and Metabolic Diseases, Normandie Université, UNIROUEN, Rouen University Hospital, Centre d’Investigation Clinique (CIC-CRB)-Inserm 1404, Rouen University Hospital, Rouen, France; 30 Department of Endocrinology-Diabetology-Nutrition, AP-HP, Avicenne Hospital, Paris 13 University, Sorbonne Paris Cité, CRNH-IdF, CINFO, Bobigny, France; 31 Equipe de Recherche en Epidémiologie Nutritionnelle (EREN), Université Sorbonne Paris Nord and Université Paris CitéInserm, INRAE, CNAM, Centre of Research in Epidemiology and StatisticS (CRESS), Bobigny, France

**Keywords:** Artificial intelligence, Cluster analysis, Continuous glucose monitoring, Diabetes complications, Glycaemia risk index, Glycaemic control, Glycaemic phenotype, Glycaemic variability, Insulin pumps, Machine learning, Type 1 diabetes

## Abstract

**Aims/hypothesis:**

Our study aims to uncover glycaemic phenotype heterogeneity in type 1 diabetes.

**Methods:**

In the Study of the French-speaking Society of Type 1 Diabetes (SFDT1), we characterised glycaemic heterogeneity thanks to a set of complementary metrics: HbA_1c_, time in range (TIR), time below range (TBR), CV, Gold score and glycaemia risk index (GRI). Applying the Discriminative Dimensionality Reduction with Trees (DDRTree) algorithm, we created a phenotypic tree, i.e. a 2D visual mapping. We also carried out a clustering analysis for comparison.

**Results:**

We included 618 participants with type 1 diabetes (52.9% men, mean age 40.6 years [SD 14.1]). Our phenotypic tree identified seven glycaemic phenotypes. The 2D phenotypic tree comprised a main branch in the proximal region and glycaemic phenotypes in the distal areas. Dimension 1, the horizontal dimension, was positively associated with GRI (coefficient [95% CI]) (0.54 [0.52, 0.57]), HbA_1c_ (0.39 [0.35, 0.42]), CV (0.24 [0.19, 0.28]) and TBR (0.11 [0.06, 0.15]), and negatively with TIR (−0.52 [−0.54, −0.49]). The vertical dimension was positively associated with TBR (0.41 [0.38, 0.44]), CV (0.40 [0.37, 0.43]), TIR (0.16 [0.12, 0.20]), Gold score (0.10 [0.06, 0.15]) and GRI (0.06 [0.02, 0.11]), and negatively with HbA_1c_ (−0.21 [−0.25, −0.17]). Notably, socioeconomic factors, cardiovascular risk indicators, retinopathy and treatment strategy were significant determinants of glycaemic phenotype diversity. The phenotypic tree enabled more granularity than traditional clustering in revealing clinically relevant subgroups of people with type 1 diabetes.

**Conclusions/interpretation:**

Our study advances the current understanding of the complex glycaemic profile in people with type 1 diabetes and suggests that strategies based on isolated glycaemic metrics might not capture the complexity of the glycaemic phenotypes in real life. Relying on these phenotypes could improve patient stratification in type 1 diabetes care and personalise disease management.

**Graphical Abstract:**

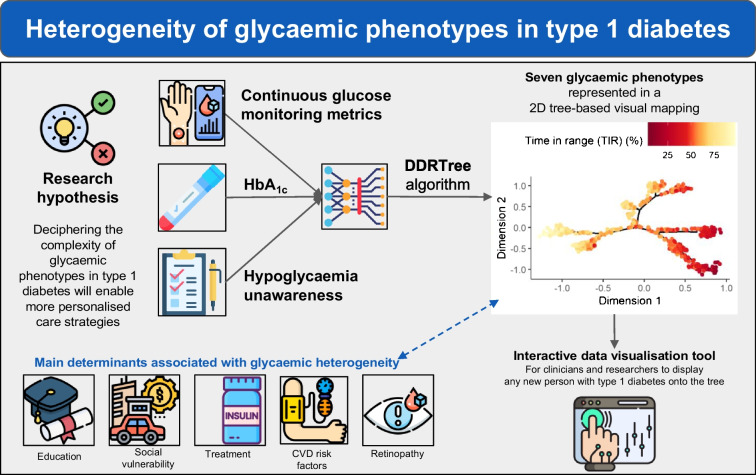

**Supplementary Information:**

The online version contains peer-reviewed but unedited supplementary material available at 10.1007/s00125-024-06179-4.


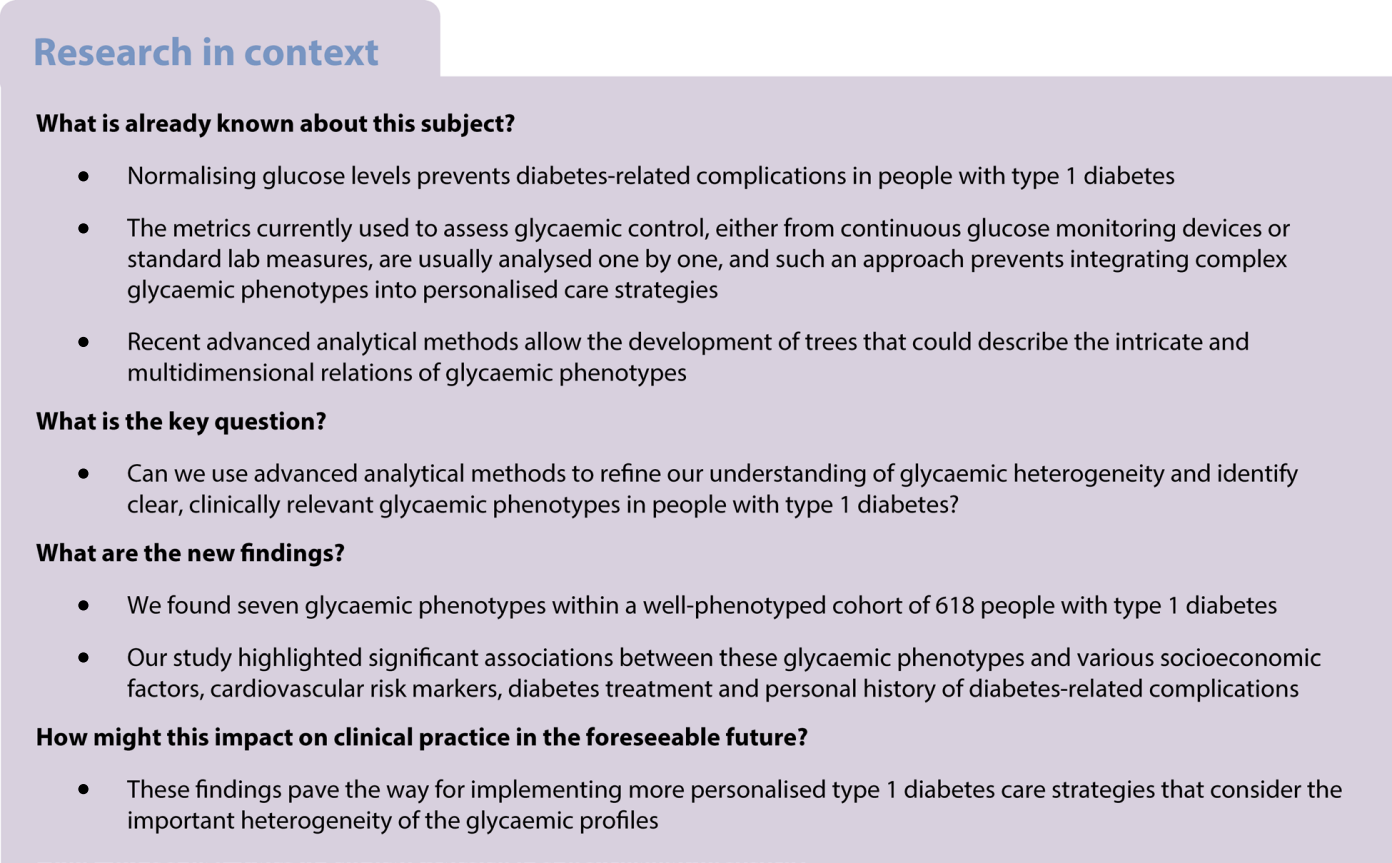


## Introduction

Over the past 25 years, the knowledge about type 1 diabetes has rapidly expanded leading to progress in clinical disease management, including continuous glucose monitoring (CGM), insulin pumps and hybrid closed-loop insulin systems (HCLs). However, type 1 diabetes remains a heterogeneous disease and wide gaps still exist in our understanding of this condition and our ability to personalise clinical care and decrease acute and chronic disease-associated complications [1]. Thus, identifying better prevention strategies based on glycaemic control is essential.

HbA_1c_ has been correlated with chronic complications [2, 3], but other aspects of glycaemic control have also been associated with these complications, such as glucose variability and hypoglycaemia [4]. Currently, in insulin-treated people, glycaemic control is mostly estimated from CGM data that assess these different components of glycaemic control [5, 6]. Among many CGM parameters, time in range (TIR) (3.9–10.0 mmol/l) is considered the most important and has been associated with various diabetes-related complications [7–9]. However, CGM devices’ information can be analysed with a global approach in a much more comprehensive fashion rather than CGM-derived metrics analysed in silo. More parameters, such as those assessing glucose variability [10] can bring complementary information and better capture the complexity of glycaemic phenotypes. In addition, the glycaemia risk index (GRI) [11, 12], a CGM-based indicator, can inform about the quality of glycaemic control, integrating a hypo- and hyperglycaemia component. Beyond the biological parameters, we must also consider individuals’ glycaemic fluctuations, such as due to hypoglycaemia unawareness, associated with severe hypoglycaemia events [13].

We can benefit from recent developments in unsupervised machine learning techniques to capture the multidimensionality of such a complex issue. Research using clustering techniques has identified clinically relevant CGM glucose patterns, mainly in individuals with type 2 diabetes and in only a small group of people living with type 1 diabetes [14]. A recent study identified 32 clinically similar clusters, where CGM profiles are homogeneous, in a large type 1 diabetes and type 2 diabetes sample [15]. However, clustering enables a mutually exclusive, discrete grouping of individuals. A continuous characterisation would describe the actual glycaemic control of the population in more detail rather than using a categorised system such as clustering. A recent study on type 2 diabetes used a tree-based approach to map diabetes phenotypes associated with diabetic complications and drug response [16]. This approach was based on data dimensionality reduction using unsupervised machine learning [17], whose main advantages were the definition of diabetes phenotypes as a continuous construct and a clinically relevant 2D visual representation.

Montaser et al proposed considering glycaemic balance in only two dimensions: exposure to hyperglycaemia and hypoglycaemia [18]. However, this 2D approach could integrate more glycaemic parameters to better understand and manage people with type 1 diabetes. A more comprehensive assessment could add additional information from a care or clinical research perspective, as an approach limited to hyperglycaemia and hypoglycaemia does not give any leverage. Reducing such a simplistic description limited to two dimensions cannot describe all phenotypes in real life.

Therefore, we hypothesised that, while keeping a 2D approach, integrating multiple glycaemic parameters into a visual representation of glycaemic phenotypes would yield more informative insights and could be more easily leveraged to enable progress in understanding real-life patients’ profiles and design personalised treatment and prevention strategies.

Our main objective was to determine glycaemic phenotypes in people with type 1 diabetes from the Study of the French-speaking Society of Type 1 Diabetes (SFDT1) and compare a tree-based approach with a more traditional clustering method. By doing so, we also aimed to identify the main determinants driving the heterogeneity of the glycaemic phenotypes.

## Methods

### Study design and population

We performed an analysis using data from SFDT1, previously described [19]. In brief, SFDT1 is an ongoing cohort study of people with type 1 diabetes in France attending hospitals or private ambulatory diabetes centres. The SFDT1 study centres cover the entire French territory, including participants of diverse ages, sexes, ethnicities and socioeconomic backgrounds. All these variables were collected except for ethnicity due to the national ethics politics in France. The SFDT1 study collects data on sex (female or male) but not on gender. The SFDT1 study includes data based on self-reported questionnaires, face-to-face interviews, physical examinations, clinical assessments, blood samples and CGM measures. The SFDT1 ClinicalTrials.gov registration no. is NCT04657783. Sanoïa provided support in the design of the study protocol, the ethical procedures and implementation of the study, and orchestrated the data collection and data-flows on its secure platform. The current analysis was performed on data collected during the baseline visit in participants enrolled between December 2020 and September 2022.

The study was approved by the ethics committee CPP Ouest V-RENNES (no. ID-RCB: 2019A01681-56) in December 2019.

We included people with type 1 diabetes aged 18 years old or more for whom demographic data were available and with CGM data from a variety of different devices, depending on which system is used by the individual user: time below range (TBR) (<3.9 mmol/l), TIR and CV. CGM data were captured within 14 days before inclusion according to the recommendations of an international consensus statement [20]. The clinical research assistants record CGM data manually from ambulatory glucose profile reports, sometimes using rounded values. Therefore, the sum of TBR, TIR and time above range (TAR) (>10 mmol/l) values was 98–102. We compared characteristics between included and not included participants.

### Variables assessing glycaemic phenotypes

We included six quantitative variables characterising complementary information on glycaemic control, glucose variability and self-perception of glycaemic fluctuations. Four variables were derived from the CGM data: TBR, TIR, CV and GRI. The CV evaluates glucose variability and is calculated as the SD/mean glucose×100 [21]. The GRI evaluates the risk of hypo- and hyperglycaemic events from four CGM variables using the formula: (3×% time spent below 3.0 mmol/l)+(2.4×% TBR)+(1.6×% time spent over 13.9 mmol/l)+(0.8×% TAR), assessing the quality of plasma glucose [11]. The last measured HbA_1c_ (mmol/mol) was the fifth variable. The sixth variable was the Gold score, which assesses hypoglycaemia unawareness and consists of a single question scored on a Likert scale from 1 (aware) to 7 (not aware) [13]. Information from TAR is redundant once TIR and TBR are included, so we omitted this information in the modelling and used it exclusively for descriptive purposes afterwards. We assessed the correlation of variables using the Spearman test.

### Variables tested as determinants of glycaemic phenotypes

We considered various individual characteristics, diabetes treatments, cardiovascular risk factors and personal history of diabetes-related complications. Individual characteristics included age, sex, diabetes duration, social vulnerability (EPICES score) [22, 23] and education level (high school diploma or more).

Diabetes treatments included the use of multiple daily injections (%), an insulin pump (%), an insulin pump combined with another device (open-loop, hypoglycaemia minimiser, sensor-augmented insulin, pump with predictive shutdown of hypoglycaemia) (%) and HCLs (%), and total daily insulin dose (U kg^−1^ day^−1^).

We included measures of cardiovascular health and cardiovascular risk factors: BMI (kg/m^2^), waist circumference (cm), systolic and diastolic blood pressure (mmHg), heart rate (beats per min [bpm]), LDL-cholesterol (mmol/l) and triglycerides (mmol/l), obesity (%) (BMI≥30 kg/m^2^), abdominal obesity (%) (waist circumference >102 cm in men and >88 cm in women) and current smoking (%).

Diabetes-related complications included retinopathy, nephropathy, neuropathy and CVD. Retinopathy diagnosis was based on patient reporting, retinography, fundus examination or ophthalmologist consultation report. Chronic kidney disease was defined as albumin/creatinine ratio >30 mg/g or eGFR <60 ml/min per 1.73m^2^ [24]. eGFR was calculated with the CKD-EPI equation [25]. Neuropathy was assessed through physical examination with the ten-item Michigan Neuropathy Screening Instrument and with a diagnosis threshold >2 [26]. CVD was defined as a clinical history of any of the following: acute coronary syndrome, angina, stroke, bypass cardiovascular surgery, coronary angioplasty, supra-aortic trunk surgery, arterial surgery of the lower limbs, non-traumatic amputation of the lower limbs, history of rhythm disturbances or hospitalisation for heart failure. Sociodemographic data, clinical data, comorbidities and complications were collected at baseline during the inclusion visit.

### Missing data management

With missing data and assuming a missing-at-random mechanism, we applied multiple imputations using the chained-equation approach and the ‘Mice’ R package [27] using R software version 4.3.2 (https://cran.r-project.org/) and RStudio software version 2023.12.1.Build 402 (https://posit.co/download/rstudio-desktop/). We imputed missing data for both the variables from which the tree originated and for covariates (continuous and categorical variable characteristics). We imputed 30 datasets (the maximum missing data observed). We applied iterations until we obtained convergence. We used an imputation model with the best predictors of missing data, including outcomes and relevant confounders. We evaluated the imputed results with means and visual representations. The continuous and categorical variables were pooled by calculating each individual’s mean and mode of the *n* imputed values, respectively, to model the phenotypic tree and the clustering analyses. To model the association of the tree’s dimensions with covariates, we pooled the estimates of all imputed datasets and calculated the CI according to Rubin’s rules.

### Statistical analysis

We described variables in the total population. We used means (SD) for continuous and normally distributed variables, median (IQR) for continuous, not normally distributed variables and count (%) for categorical variables. We compared demographic variables between included and not included participants.

#### Tree-based analysis

We preprocessed the six above-mentioned glycaemic variables plus age. We checked for skewness (i.e. skewness ≥1 with the ‘e1071’ R package) and log normalised whenever appropriate. We performed a data dimensionality reduction with Discriminative Dimensionality Reduction with Trees (DDRTree), a reversed graph embedding algorithm (Monocle R package), on the residuals of the six log-normalised variables of interest [17]. The tree’s structure resulting from the DDRTree algorithm is obtained in a fully data-driven, unsupervised manner and, as such, is not controlled by the user. The analysis projected the individuals into a 2D tree structure. This technique identifies tree branches’ starting points, representing glycaemic phenotypes’ divergences, and is analysed as a dynamic biological process. The output was the distribution of each target variable in a tree structure in a 2D reduced form. This visual structure allows us to observe a continuous distribution of the individuals along the tree observing distances (the most extreme values are kept in the distal parts of the branches, and the mixed phenotypes are observed in the proximal portions of the tree).

We fitted linear regression models between the two dimensions of the visual representation of the phenotypic tree and each of the six target variables and other covariates to identify the main determinants associated with the heterogeneity of the glycaemic phenotypes. We studied the spatial autocorrelation of each of the six variables for internal validation by plotting them with global Moran’s *I* (MI) values [28] and using the ‘sdep’ R package.

#### External validation

We performed an external validation of our DDRTree algorithm by analysing a different dataset consisting of 604 SFDT1 participants, included later in 2022 and 2023, that were not included in our initial submission. Using the same methodology, we applied the DDRTree algorithm and quantified the stability of our original findings.

Using the original dataset with 618 participants, we obtained the values for Dimension 1 (Dim1) and Dimension 2 (Dim2). Then, we performed a generalised additive model (GAM) to predict these dimensions with age, sex and the six variables of the tree as linear predictors. The GAM uses smooth functions and penalised regression splines. We used the function ‘predict’ to obtain the predicted values of dimensions in the new dataset. The first argument was the GAM model, and the second was the new data (604 new participants).

We compared the new dataset’s calculated Dim1 and Dim2 with the predicted dimensions derived from the GAM model with the original dataset, with Spearman correlations, linear regression, Bland–Altman plots and intraclass correlation coefficient (ICC).

#### Clustering analysis

We performed a parallel clustering analysis with the same age- and sex-residualised dataset we used for the trees, scaling the values. Then, we applied a principal component analysis (PCA) on the six residualised, centred variables. The PCA generated six coordinates (‘stats’ R package). We performed *k*-means with the first two coordinates of the PCA, testing from two to ten clusters.

We relied on three independent methods to choose the optimal number of clusters: the Gap statistic, the Silhouette score and the Elbow method. We assessed the cluster stability by calculating the mean Jaccard similarity coefficient (with 2000 bootstrapping). We described the sample population by clusters. We performed a multinomial logistic regression (‘nnet’ R package) to compare the characteristics of participants among clusters. We assessed the goodness of fit with the McFadden score. Values from 0.2 to 0.4 indicate a very good fit of the model. Finally, we overlaid this cluster distribution on the main phenotypic tree to compare the two methods.

### Sensitivity analysis

We investigated causation among the two dimensions and the input variables used to generate the tree with a causal Bayesian network analysis and compared it with a non-directed network analysis. We used the R packages ‘bnlearn’ and ‘bootnet’, respectively.

This study is reported following the Strengthening the Reporting of Observational Studies in Epidemiology (STROBE) guidelines. We added a glossary describing the main statistical terms (electronic supplementary material [ESM] Table 1).

## Results

### General description

We included 618 participants fulfilling the inclusion criteria (ESM Fig. 1) recruited in 20 centres in France. The 141 excluded participants showed no difference in diabetes duration. However, they were slightly younger, more frequently men, more socially vulnerable, less educated and with higher HbA_1c_ than the 618 participants (ESM Table 2). We observed missing data from 33.3% to 0% (ESM Table 3).

Table 1 outlines the population’s key characteristics. The six variables used to determine the glycaemic phenotypes were (mean [SD]): HbA_1c_ 59.4 (13.3) mmol/mol (7.6% [1.2%]), TIR 57.0% (16.4%), CV 38.6% (8.3%) and GRI 53.0 (21.6); and median (IQR): Gold score 2.0 (2.0, 3.0) per point (pp) and TBR 4.0% (2.0%, 8.9%).

**Table 1:** Population characteristics of the SFDT1 cohort (*N* available data=618)

Characteristic	Value
Individual characteristics	
Age, years	40.6 ± 14.1
Women	291 (47.1)
Diabetes duration, years	22.9 ± 14.2
Social vulnerability	143 (23.1)
Higher education	418 (67.6)
Glycaemic control	
TBR, % of time	4.0 (2.0, 8.9)
TIR, % of time	57.0 ± 16.4
TAR, % of time	36.4 ± 18.2
CV, %	38.6 ± 8.3
GRI, pp	53.0 ± 21.6
HbA_1c_, mmol/mol	59.4 ± 13.3
HbA_1c_, %	7.6 ± 1.2
Gold score, pp	2.0 (2.0, 3.0)
Diabetes treatment	
Multiple daily injections	309 (50.0)
Pump only	173 (28.0)
Pump plus other^a^	108 (17.5)
HCLs	28 (4.5)
Total insulin dose, U kg^−1^ day^−1^	0.52 ± 0.27
Cardiovascular risk factors	
BMI, kg/m^2^	25.9 ± 5.1
Obesity	106 (17.2)
Abdominal obesity	180 (29.1)
Current smoker	120 (19.4)
Systolic blood pressure, mmHg	123.6 ± 16.5
Diastolic blood pressure, mmHg	72.5 ± 11.1
Heart rate, bpm	76.7 ± 14
LDL-cholesterol, mmol/l	2.6 ± 0.9
Triglycerides, mmol/l	1.0 ± 0.6
Diabetes complications	
Retinopathy	250 (40.5)
Nephropathy	87 (14.1)
Neuropathy	296 (47.9)
CVD	48 (7.8)

Data are mean ± SD, *n* (%) or median (IQR)

SD was calculated according to Rubin’s rules for imputed data. IQR is the difference between Q3 and Q1

^a^Open-loop, hypoglycaemia minimiser, sensor-augmented insulin pump, insulin pump with predictive shutdown of hypoglycaemia

The majority of participants were male (52.9%), with a mean age of 40.6 (SD 14.1) years and a mean diabetes duration of 22.9 (SD 14.2) years. CGM sensor usage was split among Freestyle Libre 1 (63.8%), Freestyle Libre 2 (27.3%), Medtronic (6.0%) and Dexcom G6 (2.9%).

### Phenotypic tree analysis

Correlations among the six chosen variables for phenotypes ranged from 0.02 (Gold/GRI) to −0.88 (GRI/TIR) (ESM Fig. 2). TBR, HbA_1c_ and Gold score had a skewness >1; therefore, they were log normalised before analysis.

**Fig. 1:**
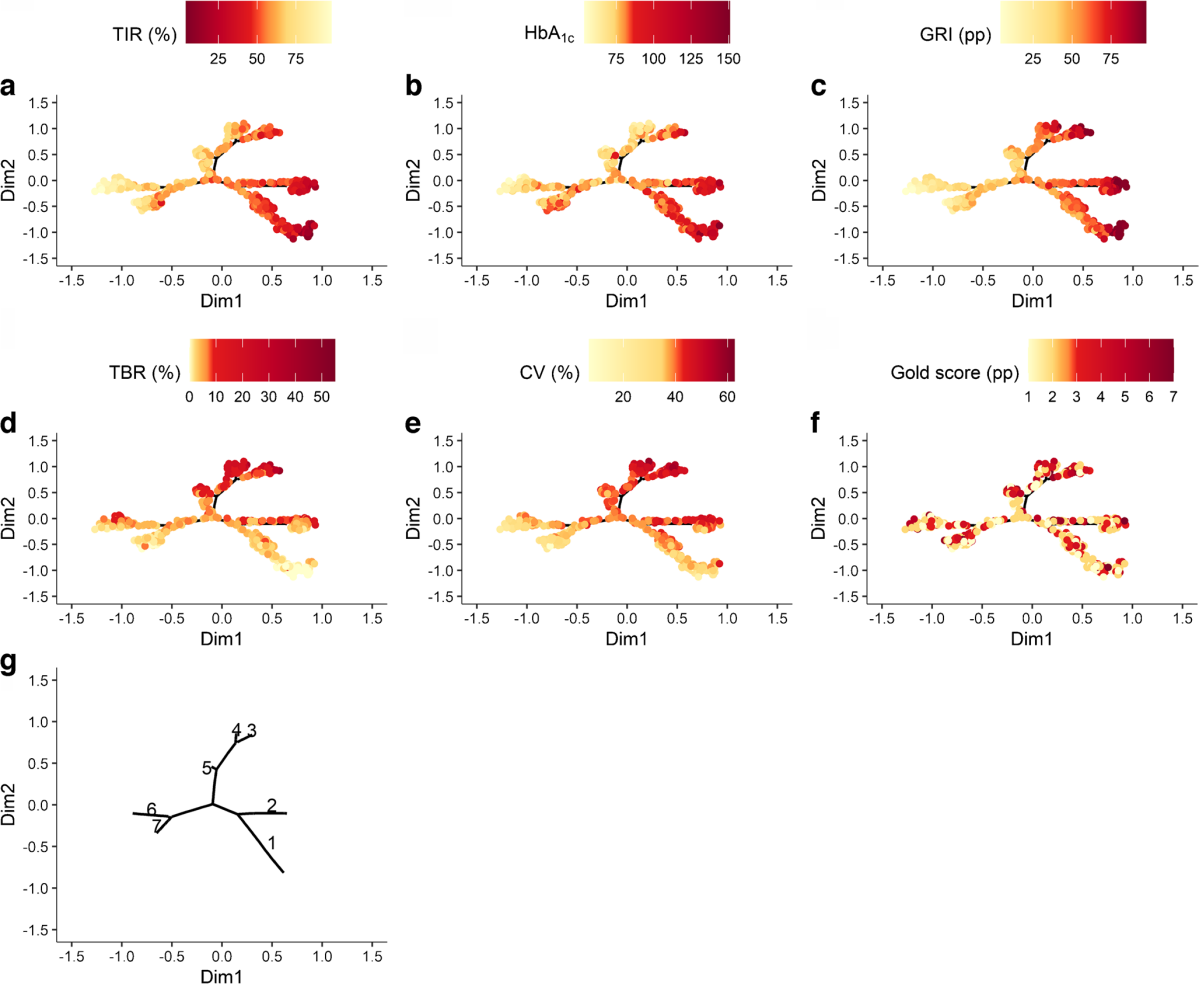
Visual representation of the heterogeneity of glycaemic control and variability in type 1 diabetes across the phenotypic tree (SFDT1 cohort, *N*=618). (**a**–**f**) Spatial distribution of glycaemic variables on the tree. (**g**) Attribution of a number to the seven glycaemic phenotypes (from 1 to 7)

Figure 1 shows the glycaemic phenotypes in a gradient-coloured tree representation. Dark red indicates less favourable values, while light yellow represents more favourable values. The extreme values are observed in the distal parts of the tree branches, and the intermediate values are in the proximal portions. We identified seven glycaemic phenotypes defined by extreme branches in distal parts of the 2D visual representation. The first glycaemic phenotype 1 in the lower-right quadrant corresponded to participants with low TIR; high HbA_1c_ and GRI; low TBR and CV; and neutral Gold score. Phenotype 2 corresponds to participants with low TIR; high HbA_1c_ and GRI; neutral TBR; high CV; and neutral Gold score. Phenotype 3 corresponds to participants with low TIR; high HbA_1c_, GRI, TBR and CV; and neutral Gold score. Phenotype 4 corresponds to participants with neutral TIR; low HbA_1c_; and high GRI, TBR, CV and Gold score. Phenotype 5 corresponds to participants with high TIR; low HbA_1c_; neutral GRI; and high TBR, CV and Gold score. Phenotype 6 corresponds to participants with high TIR; low HbA_1c_ and GRI; and neutral TBR, CV and Gold score. Phenotype 7 corresponds to participants with neutral TIR; high HbA_1c_; low GRI, TBR and CV; and neutral Gold score. Phenotypes 1 to 7 are visualised in Fig. 1 and Table 2.

**Table 2:** Characterisation of the trend of each variable within the tree in the seven phenotypes

Phenotype	TIR	HbA_1c_	GRI	TBR	CV	Gold score
1	↓↓	↑↑	↑↑	↓↓	↓	↔
2	↓↓	↑↑	↑↑	↔	↑	↔
3	↓	↑	↑↑	↑↑	↑↑	↔
4	↔	↓	↑	↑↑	↑↑	↑
5	↑	↓	↔	↑	↑	↑↑
6	↑↑	↓↓	↓↓	↔	↔	↔
7	↔	↑	↓↓	↓	↓	↔

**Fig. 2:**
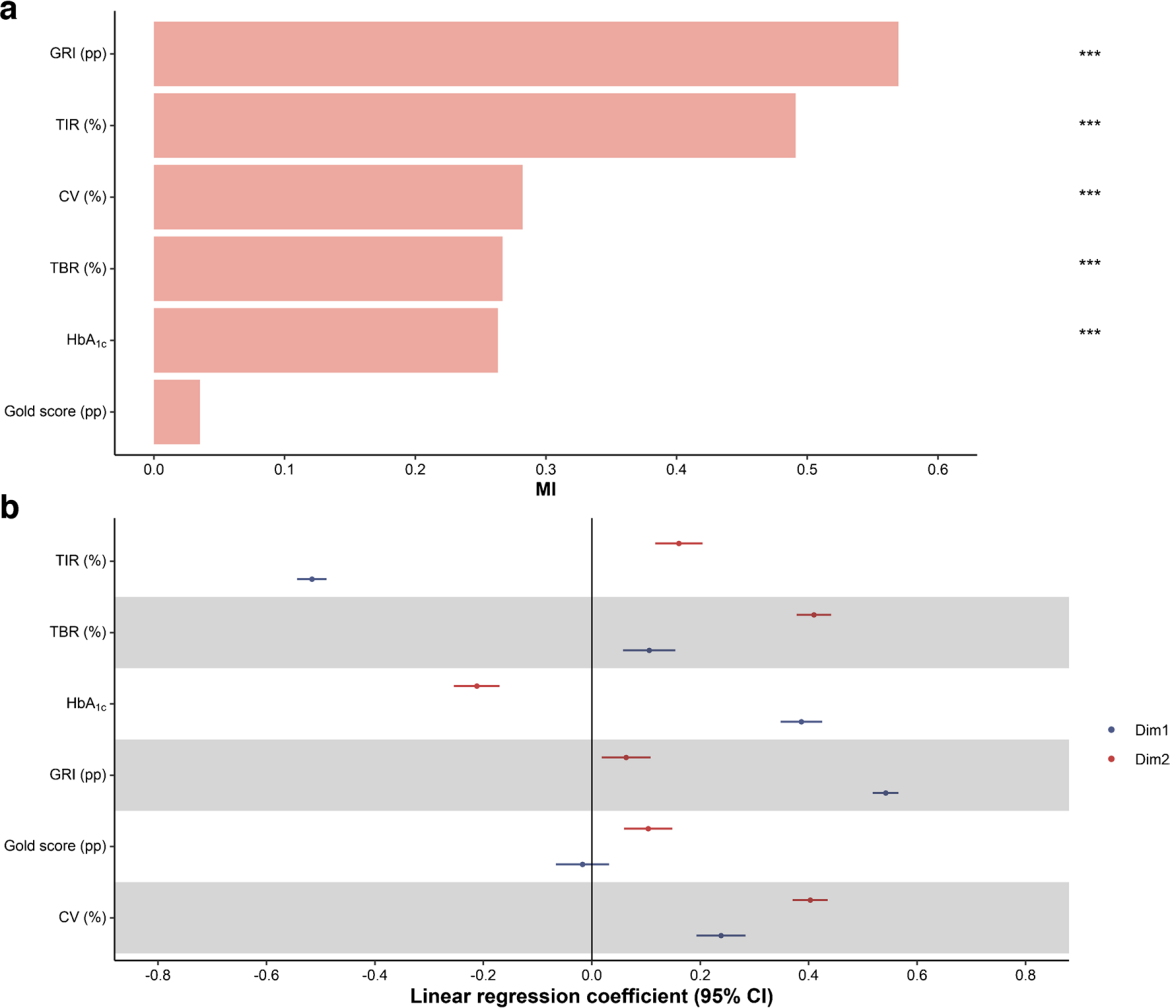
Association of glycaemic variables with the two dimensions of the tree and spatial autocorrelation MI (SFDT1 cohort, *N*=618). (**a**) MI of spatial autocorrelation of each variable. (**b**) Linear regression of glycaemic variables and the two dimensions of the tree. The blue and red lines represent the coefficient and 95% CI of Dim1 (horizontal) and Dim2 (vertical), respectively. ***p<0.001

The variables contributing the most to the phenotypic heterogeneity, as assessed by the highest spatial autocorrelation in the tree, were, in decreasing order: GRI (MI 0.57), TIR (MI 0.49), CV (MI 0.28), TBR (0.27), HbA_1c_ (MI 0.26) and the Gold score (MI 0.04) (Fig. 2a). Moderate values of all six variables characterised people with type 1 diabetes in the proximal region.

### Determinants of heterogeneity in the phenotypic tree

Overall, the 2D visual representation can be interpreted along the horizontal (Dim1) and the vertical (Dim2) dimensions. Dim1 was positively associated with elevated GRI (coefficient [95% CI]) (0.54 [0.52, 0.57]), HbA_1c_ (0.39 [0.35, 0.42]), CV (0.24 [0.19, 0.28]) and TBR (0.11 [0.06, 0.15]), and negatively with TIR (−0.52 [−0.54, −0.49]). Dim2 was positively associated with TBR (0.41 [0.38, 0.44]), CV (0.40 [0.37, 0.43]), TIR (0.16 [0.12, 0.20]), Gold score (0.10 [0.06, 0.15]) and GRI (0.06 [0.02, 0.11]), and negatively with HbA_1c_ (−0.21 [−0.25, −0.17]) (Fig. 2b).

### Potential drivers of heterogeneity of glycaemic phenotypes

**Fig. 3:**
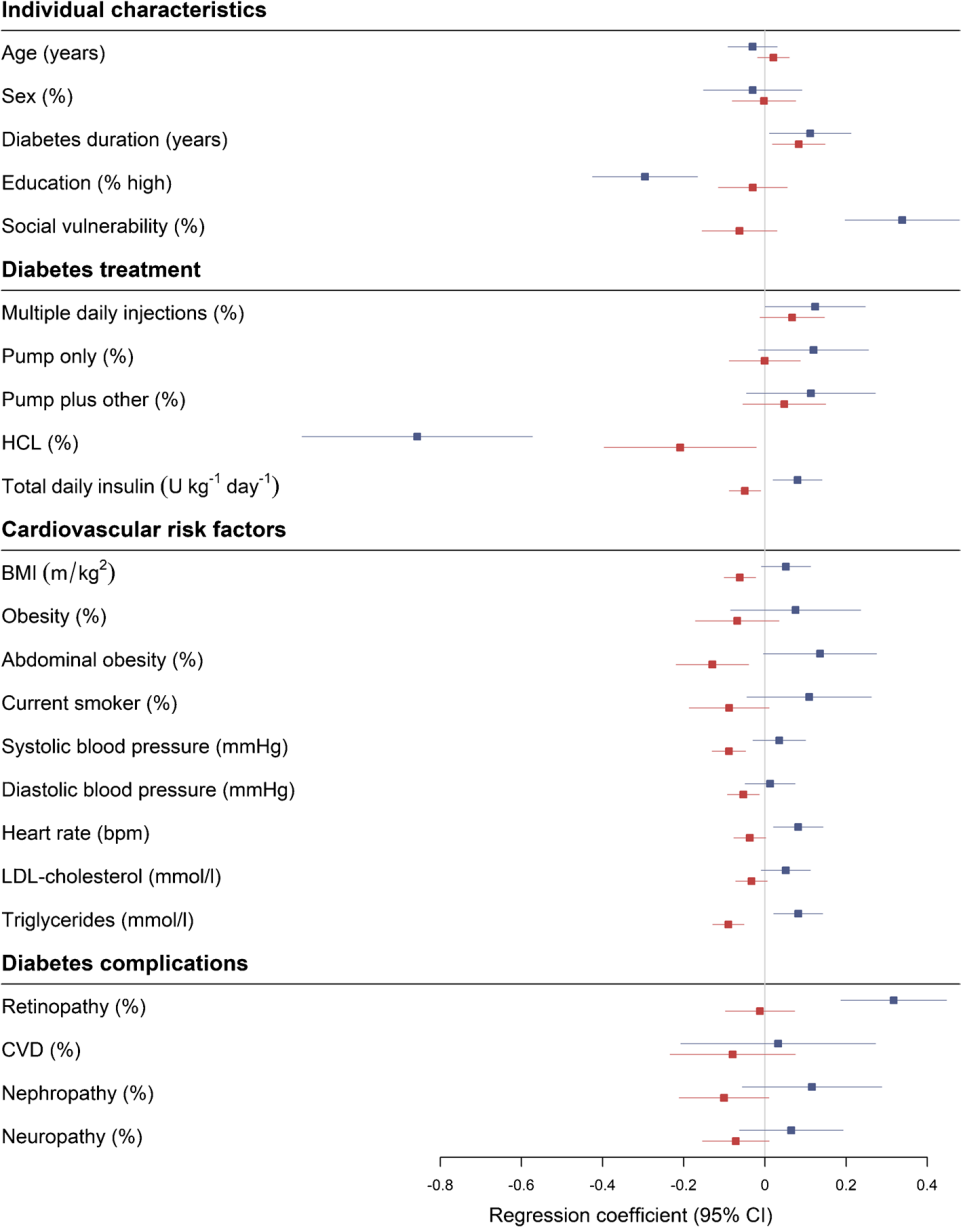
Determinants of glycaemic phenotype heterogeneity, according to the two dimensions of the phenotypic tree (SFDT1 cohort, *N*=618). Age and sex were residualised. CIs were calculated according to Rubin’s rules. Pump only: treatment with insulin pump only. Pump plus other: pump plus opened loop sensor or hypo minimiser. The blue and red lines are coefficients and 95% CI of Dim1 (horizontal) and Dim2 (vertical), respectively

Figure 3 and ESM Table 4 show that social vulnerability (coefficient [95% CI]) (0.28 [0.17, 0.40]), total daily insulin (0.07 [0.02, 0.12]), abdominal obesity (0.12 [0, 0.23]), higher heart rate (0.07 [0.02, 0.12]), triglycerides (0.08 [0.03, 0.13]) and retinopathy (0.24 [0.14, 0.35]) were positively associated with Dim1. On the other hand, using an HCL (−0.63 [−0.86, −0.40]) and higher education (−0.25 [−0.36, −0.15]) were negatively associated with Dim1.

Dim2 was positively associated with diabetes duration (0.11 [0.04, 0.19]) and negatively with HCLs (−0.29 [−0.51, −0.08]), total daily insulin (−0.06 [−0.10, −0.01]), BMI (−0.07 [−0.12, −0.03]), abdominal obesity (−0.16 [−0.26, −0.05]), systolic blood pressure (−0.09 [−0.14, −0.05]), diastolic blood pressure (−0.06 [−0.11, −0.02]) and triglycerides (−0.1 [−0.15, −0.06]).

We developed an interactive data visualisation tool that enables clinicians to enter characteristics from any individual with type 1 diabetes and project them onto the glycaemic phenotypic tree (ESM Fig. 3; online tool can be accessed at https://sfdt1.shinyapps.io/sfdt1/).

### External validation

We analysed the characteristics of the new dataset compared with the original dataset. We found that, overall, participants from the new dataset had significantly lower TBR, TIR, GRI and HbA_1c_, more frequently used pumps and automated insulin delivery systems and had less frequent retinopathy and neuropathy (ESM Table 5). When we compared agreement and associations between calculated dimensions using the original dataset and predicted dimensions using the new dataset, we observed high stability, with very strong to strong correlations (Dim1: correlation coefficient [CC] =0.92 [95% CI 0.90, 0.93], Dim2: CC=0.88 [95% CI 0.86, 0.89]), a linear regression showing significant associations (*r*^2^ Dim1=0.84, *r*^2^ Dim2=0.77) and a high ICC (Dim1: ICC=0.92 [95% CI 0.91, 0.94], Dim2: ICC=0.91 [95% CI 0.89, 0.92]) (ESM Fig. 4).

### Clustering analysis

**Fig. 4:**
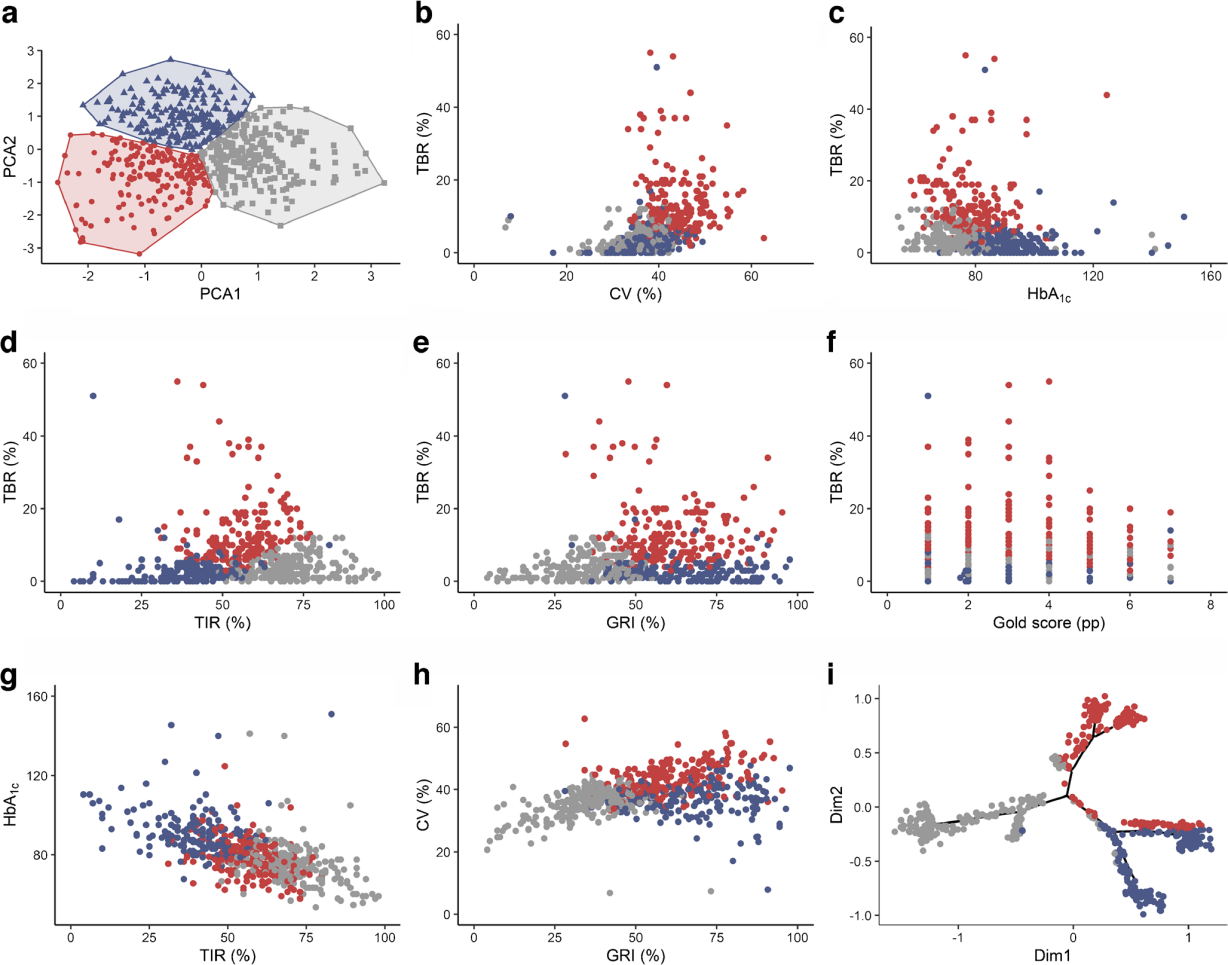
Clusters identified by *k*-means (SFDT1 cohort, *N*=618). Cluster 1 (‘Euglycaemia’), Cluster 2 (‘Hyperglycaemia’) and Cluster 3 (‘Hypoglycaemia’) are represented in grey, blue and red, respectively. (**a**) Cluster visualisation in the two PCA coordinates. (**b**–**h**) Cluster localisation in the six variables assessing glycaemic phenotypes. (**i**) Cluster distribution overlaying the glycaemic phenotypic tree. PCA1, component 1; PCA2, component 2

According to the Silhouette score, Gap statistic and Elbow method, the optimal model was composed of three clusters: ‘Euglycaemia’, Hyperglycaemia’ and ‘Hypoglycaemia’ (ESM Fig. 5). The clusters were well defined (Fig. 4) and highly stable (average Jaccard=0.96, 0.94 and 0.94 for clusters 1, 2 and 3, respectively).

A high TIR characterised the ‘Euglycaemia’ cluster (*n*=179) (mean 70.4%), and low TBR (3.8%), TAR (25.8%), CV (35.0%), HbA_1c_ (53 mmol/mol [7.0%]) and GRI (34.3) values. The ‘Hyperglycaemia’ cluster (*n*=236) was characterised by high TAR (57.3%), GRI (71.0) and HbA_1c_ (70 mmol/mol [8.6%]), and low TIR (39.9%), TBR (2.8%) and Gold score (2.3). The ‘Hypoglycaemia’ cluster (*n*=203) was characterised by high TBR (13.0%), CV (44.9%), GRI (65.4) and Gold score (2.9).

ESM Table 6 shows characteristics of ‘Hyperglycaemia’ and ‘Hypoglycaemia’ clusters compared with the ‘Euglycaemia’ cluster. ESM Fig. 6 shows differences between the ‘Hyperglycaemia’ and ‘Hypoglycaemia’ clusters compared with the ‘Euglycaemia’ cluster.

We overlaid the cluster distribution obtained with *k*-means on the glycaemic phenotypic tree structure (Fig. 4i). The ‘Hyperglycaemia’ cluster, in blue, overlapped with two distinct glycaemic phenotypes (phenotypes 1 and 2 located on the right-hand side of the panel), whereas the ‘Hypoglycaemia’ cluster, in red, was mainly present in two different sub-phenotypes in the distal upper part of the tree (phenotypes 3 and 4), but also in the glycaemic phenotypes 2 and 5. Finally, the ‘Euglycaemia’ cluster, in grey, corresponded mainly to participants situated in the glycaemic phenotypes on the left-hand side of the visual representation (phenotypes 6 and 7) and in the proximal region of the tree.

### Sensitivity analysis

ESM Fig. 7 shows a causal mediation plot. We have found that Dim2 is the most central variable in the network and that TBR and Gold score are determinants of Dim2. The GRI is a determinant of Dim1 (ESM Fig. 8).

## Discussion

This work identified a relevant visual representation of the various glycaemic phenotypes in people with type 1 diabetes. We observed a proximal region with average glycaemic values and then seven distinct glycaemic phenotypes in the distal areas of the panel. Using a method enabling continuous characterisation of the glycaemic phenotypes, as opposed to traditional discrete clustering techniques, we provided a deep phenotyping approach closer to the glycaemic heterogeneity in type 1 diabetes. Clustering methods yield results as mutually exclusive categorical groups within which substantial residual intra-variability may persist. With a tree-based approach, there is no categorisation. It is possible to position the individual in a 2D representation and, simultaneously, get highly granular information on their phenotype, thanks to its position on the tree branches. The further the individual is from the centre, the more extreme the phenotype.

The spatial distribution we found in such a 2D panel allows a better understanding of the distribution of each glycaemic variable and highlights the similarities and differences among them, as opposed to an independent interpretation of each performed separately. We have uncovered more variability or refined phenotypes with our methodology. The shape of the tree can vary depending on the input data. The dimensions, in contrast, are stable. Therefore, analysing Dim1 and Dim2 rather than the branches is preferable.

This work also highlights the relevance of the recently defined GRI [11], which showed the highest spatial autocorrelation to explain the phenotypic tree heterogeneity. This newly proposed index brings a benefit and is influential in assessing glycaemic control beyond HbA_1c_ and TIR. Its importance in evaluating glycaemic quality seems much more robust in hyperglycaemia than in hypoglycaemia. We confirmed that the GRI or its components are good descriptors for plasma glucose heterogeneity. Moreover, new evidence showed that the GRI was also associated with the demographic and socioeconomic status of people with type 1 diabetes [29]. Apart from the Gold score, information from the other glycaemic variables was found complementary, allowing us to capture more variability in the glycaemic profiles.

On top of the primary analysis, we performed a clustering analysis using the same six glycaemic variables. We identified three discrete, mutually exclusive clusters which we then labelled ‘Euglycaemia’, ‘Hypoglycaemia’ and ‘Hyperglycaemia’. These results align with previous work from Kahkoska et al, who studied 234 adolescents with type 1 diabetes and identified dysglycaemia phenotypes using clustering techniques with neural networks to determine the best number of clusters [30]. When the discrete cluster distribution was overlaid on the phenotypic tree, we noticed that the tree-based distribution enabled a more granular characterisation. For example, people in the ‘Hyperglycaemia’ cluster were in two distinct branches in the upper part of the phenotypic tree.

Similarly, the upper-left branch was shared between the ‘Euglycaemia’ and ‘Hypoglycaemia’ clusters, while the upper-right branch was shared between the ‘Hyperglycaemia’ and ‘Hypoglycaemia’ clusters. Our work suggests the benefit of using continuous approaches over traditional clustering methods. This visual approach based on dimensionality reduction and continuous distribution of different phenotypes in a tree structure was first applied by Nair et al in type 2 diabetes [16, 30]. We did not find any similar approach performed in type 1 diabetes to date.

We found that the variables were highly autocorrelated, with MI values all positive, suggesting a solid clustering of observations. The variable with the most spatial autocorrelation was the GRI (MI 0.57), while the lowest was the Gold score. Contrasting with the other variables included in the tree (numerical and continuous), the Gold score was numerical but discrete, which may explain the lack of spatial autocorrelation. In their work on type 2 diabetes, Nair et al also found positive values for MI of a magnitude similar to ours [16].

Our analysis showed heterogeneous glycaemic phenotyping in type 1 diabetes visualised as a continuous gradient in the distribution of observations on the tree, as found with type 2 diabetes [16]. The analyses were performed using age- and sex-residualised variables, enabling the discovery of glycaemic phenotypes beyond these standard clinical variables. By considering that age could explain some heterogeneity, we also indirectly minimised the impact of diabetes duration on defining the glycaemic phenotypes.

Dim1 was associated with social vulnerability and lower education. This aligns with previous works showing that lower education and health literacy could hinder diabetes care [16, 31]. Moreover, other barriers exist to diabetes technology adoption from users and providers, such as physical burdens, expectations, provider education and costs [32]. Socioeconomic factors have already been associated with suboptimal glycaemic control [32–35]. The observed association of social vulnerability with glycaemic phenotypes (Dim1) may be mediated by different access to innovative care (such as automated insulin delivery systems), lower diabetes education and diabetes self-management skills [36].

We did not find any association between Dim2 and social vulnerability, which aligns with previous research findings [37, 38]. Hypoglycaemia is likely driven by other mechanisms not directly associated with social vulnerability [39].

We found that HCL and CGM devices were inversely associated with Dim1 and Dim2. These findings are consistent with RCTs showing increased TIR and decreased TBR, CV and GRI when using HCLs [40, 41].

Retinopathy was associated with Dim1. Similarly, El Malahi et al studied 515 individuals with type 1 diabetes and found that microvascular complications were associated with lower TIR and higher HbA_1c_ but did not find any association with CVD [42]. Mesa et al performed a cross-sectional analysis in 152 individuals with CVD, finding associations between reduced TIR and microvascular disease and between increased TBR and carotid plaques [43]. In addition, there is evidence that retinopathy usually precedes diabetic nephropathy [44]. We did not find any association of nephropathy, neuropathy or CVD with either dimension, probably because the main drivers of these complications are age dependent, and the variables used to build the tree were residualised for age and sex.

The sensitivity analysis results allow us to observe how crucial dimensions are in the network and suggest some hypotheses for causality that should be confirmed in longitudinal studies.

Our study has some limitations. The glycaemic phenotypes were based on glycaemic markers and CGM-derived measures. Excluding non-CGM users could have introduced selection bias, and our findings may not be directly extrapolated to people with type 1 diabetes not using CGM devices. Although CGM is reimbursed in France [6], it is likely that excluding the non-CGM users of the SFDT1 study population has resulted in a reduced variability of the observed glycaemic phenotypes [45]. In addition, CGM was collected simultaneously with clinical data, which can further limit the interpretation of the results. We observed missing data for some covariates. However, we applied multiple imputations with a state-of-the-art method to avoid introducing bias, as if we had performed complete data analysis. Our study is cross-sectional, and we cannot infer causality. Finally, we analysed only sex and not gender data. This lack of analysis may affect the generalisability of our results to individuals with different gender identifications of biological sex.

On the other hand, this study also has several strengths. Using an innovative unsupervised machine learning technique, we analysed a large group of deeply characterised participants from the SFDT1 study. This technique enabled a continuous and visual representation of the complexity in the glycaemic phenotypes in people with type 1 diabetes and, as such, will contribute to a more personalised diabetes medicine [46]. This analysis provides, for the first time, a comprehensive description of the glycaemic phenotypes in real life, as opposed to controlled settings from RCTs.

We also provided an online, user-friendly data visualisation tool, where clinicians can project a person with type 1 diabetes within the phenotypic tree for illustrative and educational purposes.

Along with a prospective analysis in the SFDT1 cohort study, such glycaemic phenotypes will have to be further analysed concerning the incidence of diabetes-related complications to demonstrate their clinical utility for diabetes care and tertiary prevention strategies. This work should now be replicated in other prospective studies to evaluate whether the glycaemic profile predicts outcomes in people with type 1 diabetes, in particular when automated insulin delivery systems are more generalised.

### Conclusion

This work provides a deep understanding of the heterogeneity of glycaemic phenotype in people with type 1 diabetes and its real-life determinants. Our results can help clinicians and researchers better understand the complexity of type 1 diabetes glycaemic phenotypes, which may be used to design future precision diabetes interventions.

### Supplementary Information

Below is the link to the electronic supplementary material.

ESM (PDF 1.85 MB)

## Data Availability

Data used for this analysis are available for academic researchers on request submitted to the scientific committee of SFDT1: cohorte.sfdt1@gmail.com. Codes created for this analysis are available on request to the corresponding author: guy.fagherazzi@lih.lu.

## Abbreviations

bpm: Beats per min
CC: Correlation coefficient
CGM: Continuous glucose monitoring
DDRTree: Discriminative Dimensionality Reduction with Trees
Dim1: Dimension 1
Dim2: Dimension 2
GAM: Generalised additive model
GRI: Glycaemia risk index
HCL: Hybrid closed-loop insulin system
ICC: Intraclass correlation coefficient
MI: Moran’s *I*
PCA: Principal component analysis
pp: per point
SFDT1: Study of the French-speaking Society of Type 1 Diabetes
TAR: Time above range
TBR: Time below range
TIR: Time in range
