# Supplementary material for: Heterogeneity of glycaemic phenotypes in type 1 diabetes
Source: Diabetologia. 2024 May 23;67(8):1567–81. doi: 10.1007/s00125-024-06179-4 (PMC11343912; doi:10.1007/s00125-024-06179-4)
Supplement: Supplementary file 1 — ESM (PDF 1.85 MB) [file 125_2024_6179_MOESM1_ESM.pdf]

ELECTRONIC SUPPLEMENTARY MATERIAL (ESM)

Contents

ESM Table 1. Glossary of statistical terms 2

ESM Table 2. Population characteristics between included and excluded participants 3

ESM Table 3. Missing data 4

ESM Table 4. Determinants of glycaemic phenotypes heterogeneity, according to the two dimensions of the phenotypic tree (SFDT1 Cohort, N=618). Linear regression values 5

ESM Table 5. External validation. Characteristics of the two populations: original dataset (n=618) and new dataset (n=604) 6

ESM Table 6. Characteristics of hyperglycaemia and hypoglycaemia clusters compared with euglycaemia cluster as the reference (SFDT1 Cohort, N=618). Multinomial logistic regression. 7

ESM Fig. 1. Inclusion flowchart 8

ESM Fig. 2. Spearman's correlation matrix of the six variables used to define the glycaemic phenotypes 9

ESM Fig. 3. Online data visualisation tool 10

ESM Fig. 4. External validation. Agreement measures between calculated and predicted dimensions among the two datasets 11

ESM Fig. 5. Optimal number of clusters 12

ESM Fig. 6. Variables associated with clusters 13

ESM Fig. 7. Bayesian network plot 14

ESM Fig. 8. Network plot 15

ESM Table 1. Glossary of statistical terms

Term	Abbreviation	Details	R package
Discriminative Dimensionality Reduction with Trees	DDRTree	Reduction dimensionality algorithm for cell trajectory analysis	Monocle
Spatial Autocorrelation (Global Moran's I)	MI	Measure of spatial autocorrelation, which are multidimensional and multidirectional. The pattern can be clustered, dispersed or random. Positive values indicates tendency of clustering	sdep
Principal Component Analysis	PCA	Reduction dimensionality algorithm	stats
K-Means Clustering	K-means	Unsupervised machine learning algorithm that identifies clusters of data points with similarities.	stats
Gap Statistic	-	Method to determine the optimal number of clusters. Compare the total intra-group variation for different values of k with their expected values with Monte Carlo simulations.	cluster
Average Silhouette Method	Silhouette score	Method to determine the optimal number of clusters. It measures the quality of a clustering by comparing the average silhouette observations for different values of k.	cluster
Elbow Method.	Elbow	Method to determine the optimal number of clusters. It finds the k value where the total intracluster variation is minimised	stats
Jaccard Similarity Coefficient	Jaccard	Method to assess the cluster stability by comparing members of two groups and determining which of them are shared or distinct. It uses bootstrapping.	fpc
Bayesian Networks	-	A directed acyclic graph or probabilistic graphical models representing the dependency of a set of variables and a global probability distribution. The objective is to determine the conditional independence of the variables.	bnlearn
Network analysis	-	Graphical representation of dependencies among a set of variables. They are not causal models per se. It allows an understanding of the interactions in a network and the most important features influencing the network.	bootnet, qgraph

ESM Table 2. Population characteristics between included and excluded participants

	Included (N=618)	Not included (N=141)
Age, years (mean (SD))	40.6 (±14.1)	38.3 (±14.9)*
Women, N (%)	291 (47.1%)	124 (39.4%)*
Diabetes duration, years (mean (SD))	22.8 (±14.1)	22.5 (±13.8)
Social vulnerability, N (%)	147 (23.4%)	167(53.0%)*
Higher education, N (%)	421 (67.0%)	151 (47.9%)*
Current smoker (%)	125 (21.3%)	38 (26.2%)
HbA_{1c}, mmol/mol (mean (SD))	59.3 (±13.0)	68.6 (±16.3)*
HbA_{1c}, % (mean (SD))	7.6 (±1.3)	8.8 (±1.5)*
BMI, kg/m^2 (mean (SD))	25.9 (±4.8)	26.8 (±4.6)

*P value <0.05

ESM Table 3. Missing data

Variable name	Number of missing	%
Age	0	0
Sex	0	0
TBR	0	0
TIR	0	0
TAR	0	0
Type of device	0	0
Diabetes duration	15	2.4
Smoking status	18	2.9
CVD	18	2.9
Higher education	18	2.9
Treatment	19	3.1
Total insulin dose	24	3.9
Retinopathy	25	4.0
HbA_{1c}	41	6.6
BMI	42	6.8
Systolic/diastolic blood pressure	42	6.8
Heart rate	42	6.8
Time above 13.9 mmol/l	43	7.0
Neuropathy	51	8.3
eGFR	57	9.2
Gold score	60	9.7
Time below 3.0 mmol/l	64	10.4
Triglycerides	74	12.0
LDL-cholesterol	77	12.5
GRI	90	14.6
Social vulnerability	100	16.2
Albuminuria	142	23.0
CV	152	24.6
Waist circumference	206	33.3

ESM Table 4. Determinants of glycaemic phenotypes heterogeneity, according to the two dimensions of the phenotypic tree (SFDT1 Cohort, N=618). Linear regression values

	Dimension 1		Dimension 2	
Variable	Coefficient (LCI, UCI)	R^2	Coefficient (LCI, UCI)	R^2
Age (years)	-0.022 (-0.072, 0.027)	0.002	0.023 (-0.022, 0.069)	0.002
Sex (%)	-0.027 (-0.126, 0.071)	0.002	0.008 (-0.083, 0.099)	0.002
Diabetes duration (years)	0.068 (-0.014, 0.150)	0.006	0.112 (0.036, 0.187)	0.015
Higher education (% high)	-0.252 (-0.357, -0.147)	0.037	-0.051 (-0.150, 0.047)	0.003
Social vulnerability (%)	0.283 (0.169, 0.397)	0.039	-0.065 (-0.173, 0.042)	0.004
Multiple daily injections (%)	0.099 (-0.001, 0.199)	0.008	0.087 (-0.005, 0.180)	0.007
Pump only (%)	0.100 (-0.010, 0.210)	0.007	-0.001 (-0.103, 0.101)	0.002
Pump plus other (%)	0.113 (-0.015, 0.242)	0.007	0.055 (-0.064, 0.174)	0.003
HCL (%)	-0.633 (-0.864, -0.402)	0.047	-0.294 (-0.512, -0.077)	0.013
Total insulin dose, U kg^{-1} day^{-1} (mean) (SD))	0.070 (0.021, 0.119)	0.015	-0.058 (-0.104, -0.013)	0.012
BMI (kg/m²)	0.047 (-0.002, 0.097)	0.008	-0.073 (-0.119, -0.028)	0.018
Obesity (%)	0.085 (-0.045, 0.215)	0.005	-0.090 (-0.210, 0.030)	0.005
Abdominal obesity (%)	0.117 (0.004, 0.230)	0.009	-0.157 (-0.261, -0.052)	0.016
Current smoker %)	0.104 (-0.020, 0.228)	0.006	-0.100 (-0.215, 0.015)	0.006
Systolic blood pressure (mm Hg)	0.043 (-0.010, 0.096)	0.006	-0.095 (-0.143, -0.047)	0.025
Diastolic blood pressure (mm Hg)	0.014 (-0.036, 0.064)	0.002	-0.064 (-0.110, -0.018)	0.014
Heart rate (bpm)	0.065 (0.016, 0.115)	0.013	-0.039 (-0.085, 0.007)	0.006
LDL-cholesterol (mmol/l)	0.041 (-0.008, 0.090)	0.006	-0.038 (-0.083, 0.008)	0.006
Triglycerides (mmol/l)	0.079 (0.030, 0.128)	0.018	-0.102 (-0.146, -0.057)	0.033
Retinopathy (%)	0.243 (0.137, 0.349)	0.034	0.012 (-0.087, 0.112)	0.002
CVD (%)	0.040 (-0.154, 0.235)	0.002	-0.096 (-0.275, 0.084)	0.003
Nephropathy (%)	0.089 (-0.051, 0.228)	0.004	-0.110 (-0.239, 0.019)	0.006
Neuropathy (%)	0.064 (-0.040, 0.168)	0.004	-0.077 (-0.173, 0.019)	0.006

Age and sex were residualised. Abbreviations: LCI: 95% lower CI; UCI: 95% upper CI. HCL: Hybrid closed-loop insulin system

ESM Table 5. External validation. Characteristics of the two populations: original dataset (n=618) and new dataset (n=604)

	Original dataset (n=618)	New dataset (n=604)	P value
Individual characteristics			
Age, years (mean (SD))	40.6 (± 14.1)	40.9 (± 14.1)	0.648
Women, N (%)	291 (47.1%)	304 (50.3%)	0.282
Diabetes duration, years (mean (SD))	22.9 (± 14.2)	23.3 (± 13.8)	0.524
Social vulnerability, N (%)	143 (23.1%)	136 (22.5%)	0.253
Higher education, N (%)	418 (67.6%)	367 (60.8%)	0.042
Glycaemic control			
TBR, % of time (median (IQR))	4.0 (2. 0, 8.9)	2.9 (1,0, 5.0)	<0.001
TIR, % of time (mean (SD))	57 (± 16.4)	59.2 ± 16.7	0.024
TAR, % of time (mean (SD))	36.4 (± 18.2)	36.9 ± 17.4	0.662
CV, % (mean (SD))	38.6 (± 8.3)	37.9 ± 8.6	0.055
GRI, pp (mean (SD))	53 (± 21.6)	49 (± 22.3)	<0.001
HbA_{1c}, mmol/mol, (mean (SD))	59.4 (±13.3)	57.8 (11.0)	0.023
HbA_{1c}, %, (mean (SD))	7.6 (± 1.2)	7.4 (± 1.0)	0.023
Gold score, pp (median (IQR))	2.0 (2.0, 3.0)	2.5 (± 1.9)	0.132
Diabetes treatment			
Multiple daily injections, N (%)	309 (50.0%)	179 (29.6%)	<0.001
Pump only, N (%)	173 (28.0%)	126 (20.9%)	0.006
Pump plus other, N (%)	108 (17.5%)	174 (28.8%)	<0.001
HCL, N (%)	28 (4.5%)	125 (20.7%)	<0.001
Total insulin dose, U kg^{-1} day^{-1} (mean (SD))	0.52 (± 0.27)	0.59 (± 0.39)	<0.001
Cardiovascular risk factors			
BMI, kg/m^2 (mean (SD))	25.9 (± 5.1)	26.4 (± 5.4)	0.175
Obesity, N (%)	106 (17.2%)	123 (20.4%)	0.482
Abdominal obesity, N (%)	180 (29.1%)	203 (33.6%)	0.169
Current smoker, N (%)	120 (19.4%)	114 (18.9%)	0.65
Systolic blood pressure, mm Hg, (mean (SD))	123.6 (± 16.5)	124.4 (± 16.2)	0.247
Diastolic blood pressure, mm Hg, (mean (SD))	72.5 (± 11.1)	72.5 (± 11.2)	0.534
Heart rate, BPM (mean (SD))	76.7 (± 14)	75.1 (± 13.8)	0.098
LDL-cholesterol, mmol/l (mean (SD))	2.6 (± 0.9)	2.4 (± 0.9)	<0.001
Triglycerides, mmol/l (mean (SD))	1.0 (± 0.6)	0.9 (± 0.6)	0.073
Diabetes complications			
Retinopathy, N (%)	250 (40.5%)	199 (32.9%)	0.013
Nephropathy, N (%)	87 (14.1%)	76 (12.6%)	0.124
Neuropathy, N (%)	296 (47.9%)	209 (34.6%)	<0.001
CVD, N (%)	48 (7.8%)	55 (9.1%)	0.398

Abbreviations: HCL: Hybrid closed-loop insulin system

ESM Table 6. Characteristics of hyperglycaemia and hypoglycaemia clusters compared with euglycaemia cluster as the reference (SFDT1 Cohort, N=618). Multinomial logistic regression.

	Cluster hyperglycaemia	Cluster hypoglycaemia	
Variable	OR (LCI, UCI)	OR (LCI, UCI)	McFadden pseudo R^2
Age (years)	1.0 (1.0, 1.0)	1.0 (1.0, 1.0)	0.001
Sex (%)	0.9 (0.6, 1.3)	0.9 (0.6, 1.3)	0
Diabetes duration (years)	1.0 (1.0, 1.0)	1.0 (1.0, 1.0)	0.001
Higher education (% high)	0.5 (0.3, 0.8)	0.5 (0.3, 0.8)	0.008
Social vulnerability (%)	3.7 (2.3, 6.1)	3.7 (2.3, 6.1)	0.023
Multiple daily injections (%)	2.0 (1.2, 3.1)	2.0 (1.2, 3.1)	0.009
Pump only (%)	1.8 (1.1, 3.1)	1.8 (1.1, 3.1)	0.006
Pump plus other (%)	2.2 (1.4, 3.5)	2.2 (1.4, 3.5)	0.011
Closed loop system (%)	0.1 (0.0, 0.2)	0.1 (0.0, 0.2)	0.088
Total daily insulin (U kg^{-1} day^{-1})	2.8 (1.3, 6.3)	2.8 (1.3, 6.3)	0.006
BMI (kg/m^2)	1.0 (1.0, 1.0)	1.0 (1.0, 1.0)	0.002
Obesity (%)	1.3 (0.8, 2.0)	1.3 (0.8, 2.0)	0.003
Abdominal obesity (%)	1.1 (0.7, 1.7)	1.1 (0.7, 1.7)	0.001
Current smoker %)	2.3 (1.3, 3.9)	2.3 (1.3, 3.9)	0.009
Systolic blood pressure (mm Hg)	1.0 (1.0, 1.0)	1.0 (1.0, 1.0)	0
Diastolic blood pressure (mm Hg)	1.0 (1.0, 1.0)	1.0 (1.0, 1.0)	0.002
Heart rate (BPM)	1.0 (1.0, 1.0)	1.0 (1.0, 1.0)	0.013
LDL-cholesterol (mmol/l)	1.0 (0.7, 1.3)	1.0 (0.7, 1.3)	0.005
Triglycerides (mmol/l)	1.4 (0.9, 2.1)	1.4 (0.9, 2.1)	0.003
Retinopathy (%)	1.6 (1.1, 2.5)	1.6 (1.1, 2.5)	0.004
CVD (%)	0.8 (0.4, 1.7)	0.8 (0.4, 1.7)	0
Nephropathy (%)	1.3 (0.7, 2.2)	1.3 (0.7, 2.2)	0.001
Neuropathy (%)	0.6 (0.4, 1.0)	0.6 (0.4, 1.0)	0.004

ESM Fig. 1. Inclusion flowchart

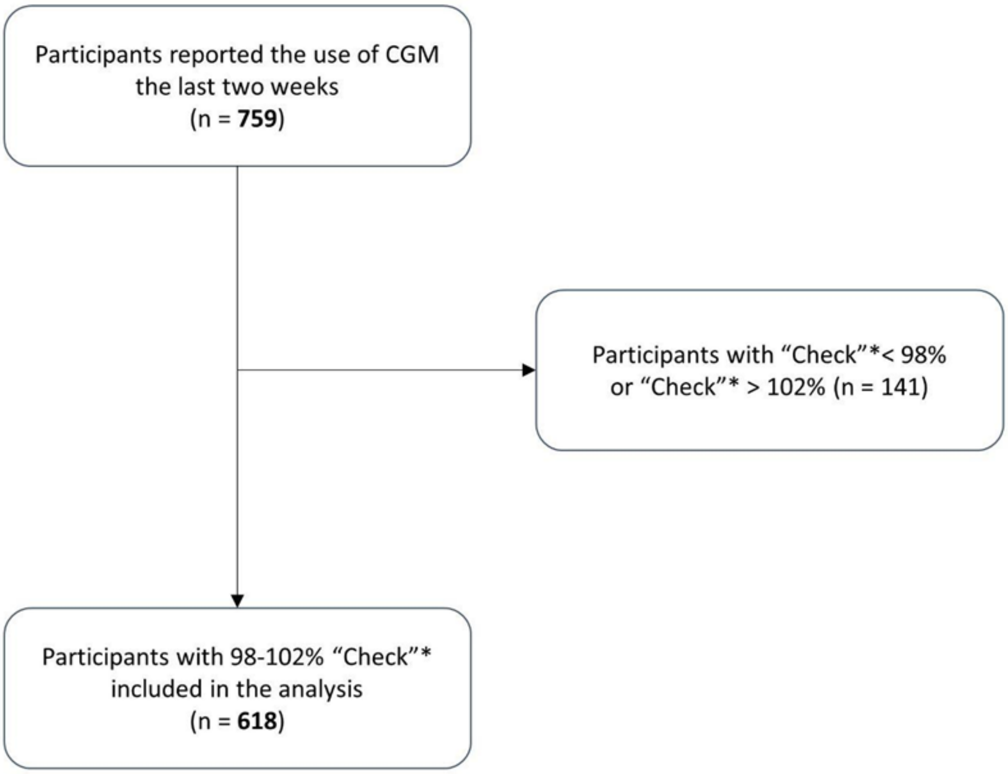

*Check = % time spent < 3.9 mmol/l+ % TIR (between 3.9 and 10 mmol/l) + % time spent > 10 mg/dl

ESM Fig. 2. Spearman's correlation matrix of the six variables used to define the glycaemic phenotypes

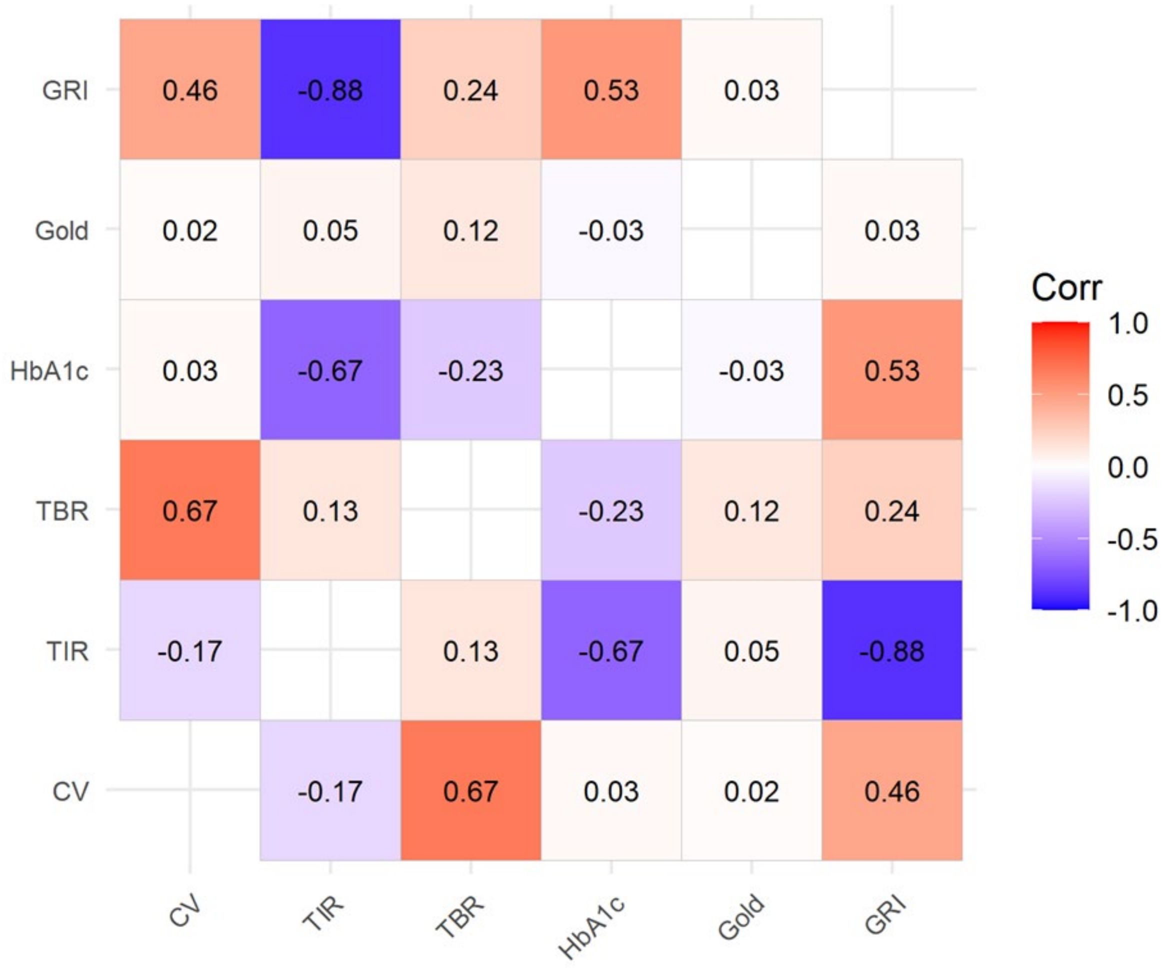

Labels: GR: Glycaemia risk index; Gold: Gold score; TBR: TBR; TIR: TIR; CV: coefficient of variation.

ESM Fig. 3. Online data visualisation tool

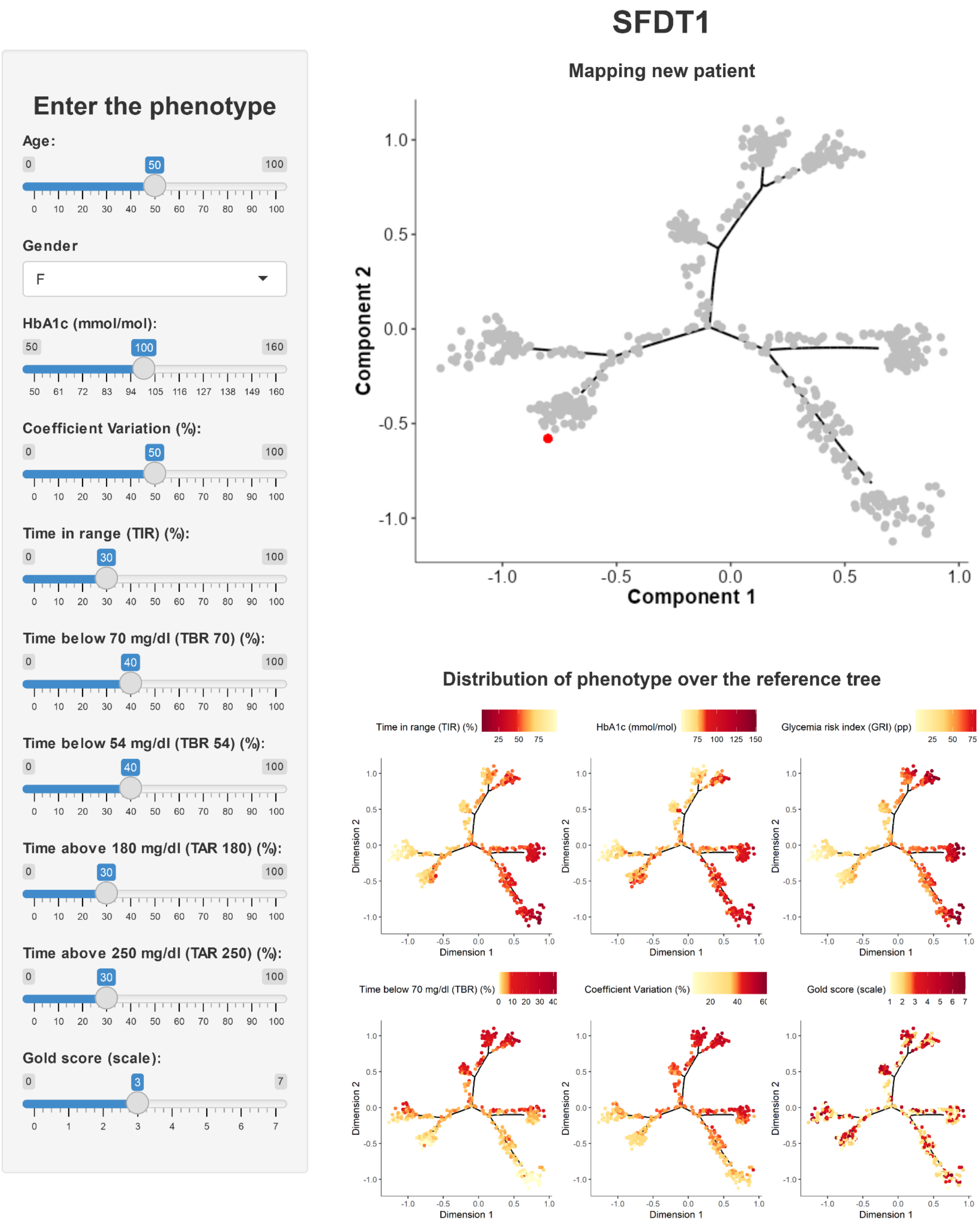

To map individuals with new people with type 1 diabetes to the generated tree, we used the mapping function described by Nair et al. [16]. Briefly, two generalised additive models were trained to predict the 2 DDRtree dimensions (the coordinates of the tree) respectively by using the SFDT1 data of 8 variables, including age, sex and the six variables of interest. Given the eight variables for a new individual, the trained model predicts the two dimensions for the individual. Then, we estimated the Euclidean distance between the predicted coordinates of the individual and all the coordinates in the generated SFDT1 tree. After that, we re-assigned the coordinates in the SFDT1 tree to the new individual with the shortest distance to the predicted points. The online tool can be accessed here: https://sfdt1.shinyapps.io/sfdt1/. The red dot corresponds to the projection of the given patient on the phenotypic tree.

ESM Fig. 4. External validation. Agreement measures between calculated and predicted dimensions among the two datasets

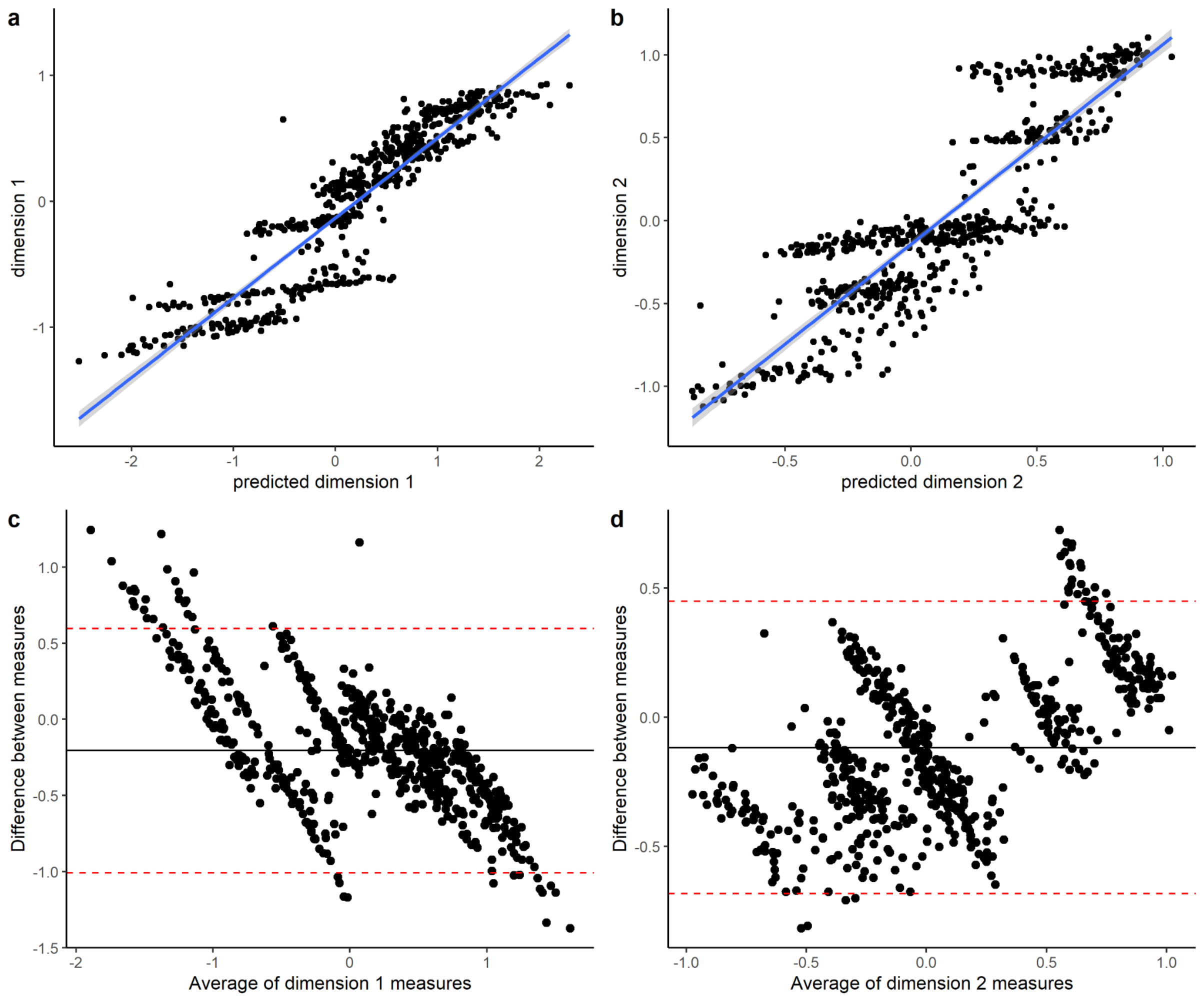

Dimension	Correlation (95% CI)	Coefficient lm (95% CI)	Adj. r^2	ICC (95% CI)
Dimension 1	0.92 (0.90, 0.93)	0.63 (0.61, 0.66)	0.84	0.92 (0.91, 0.94)
Dimension 2	0.88 (0.86, 0.89)	1.20 (1.15, 1.26	0.77	0.91 (0.89, 0.92)

Panels A and B show linear regressions. The dependent variable was the calculated dimension and the independent variable was the predicted dimension. The actual values for the domains were obtained after performing DDRTree on the original dataset. The predicted dataset was based on a GAM model with actual dimensions from the new dataset. The table shows the following association-agreement metrics between calculated and predicted dimensions: Spearman correlation (Correlation), linear regression coefficient (Coefficient lm) with its 95% CI (95% CI), adjusted R squared (Adj. r^2.) and intraclass correlation coefficient (ICC) with its 95% CI.

ESM Fig. 5. Optimal number of clusters

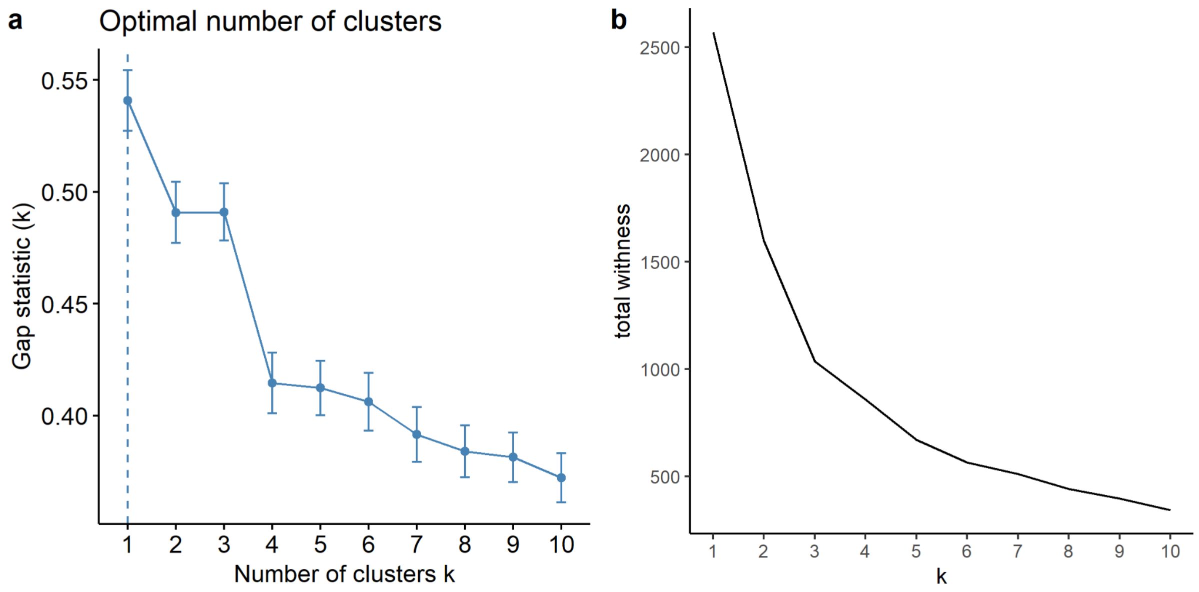

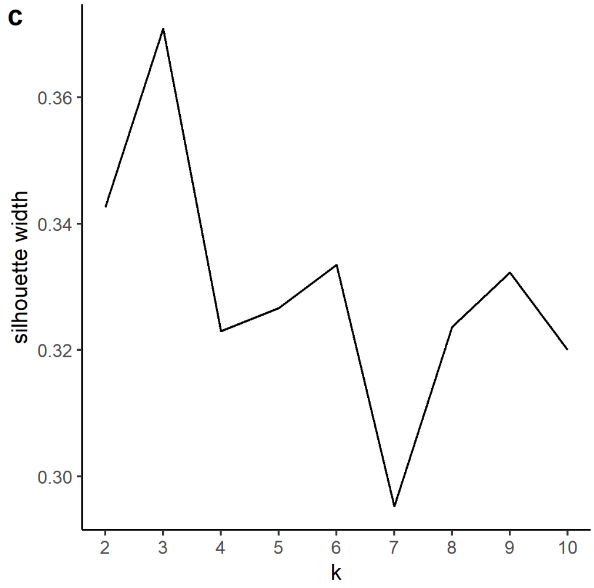

d

Number of Clusters	Silhouette score
2	0.34
3	0.37
4	0.32
5	0.33
6	0.33
7	0.3
8	0.32
9	0.33
10	0.32

Panel A: Gap statistics; Panel B: Elbow; Panel C and D: Silhouette scores. An optimal number of clusters was found to be equal to 3.

ESM Fig. 6. Variables associated with clusters

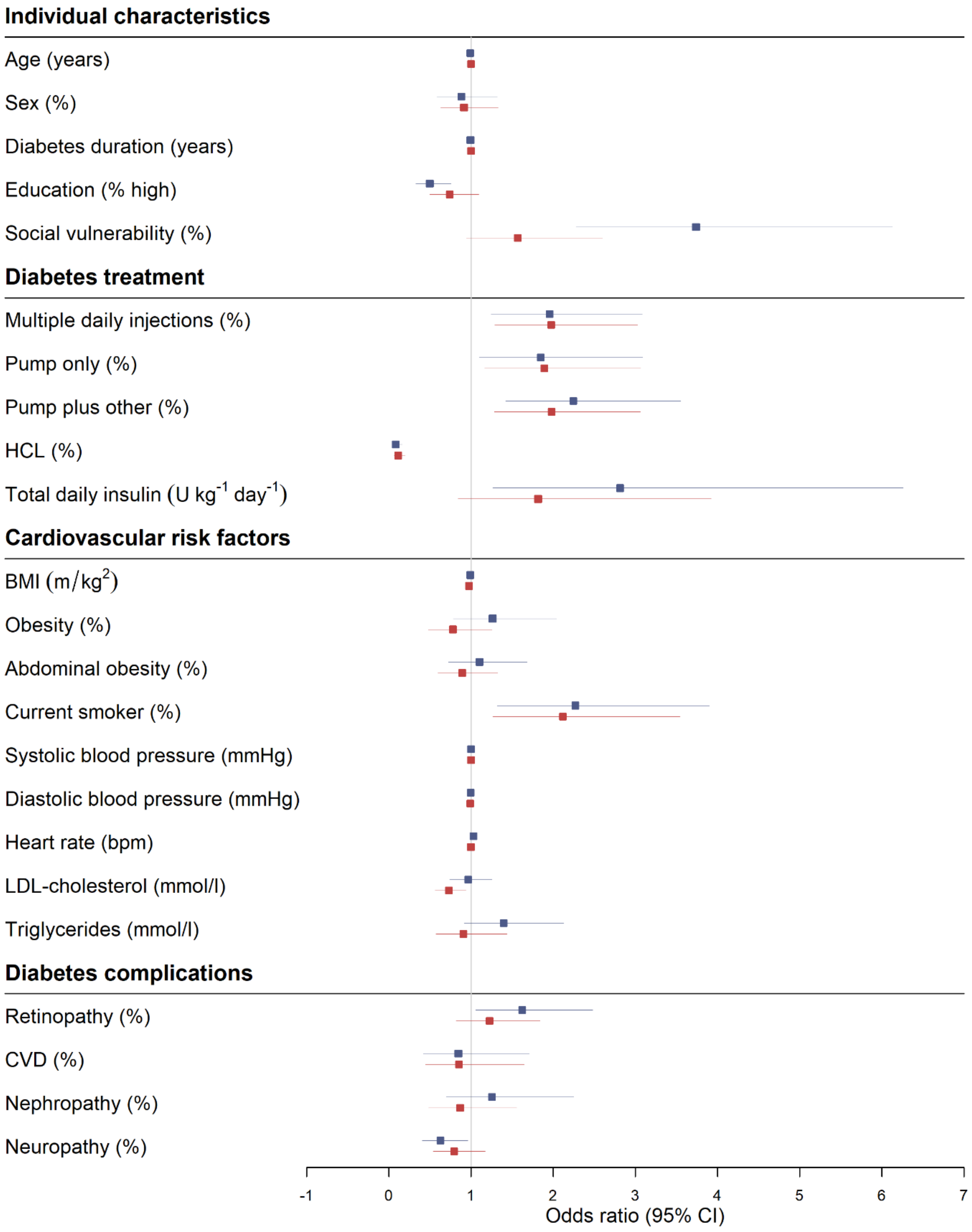

CI were calculated according to Rubin's rules. Abbreviations: Pump only: Treatment with insulin pump only. Pump plus other: Pump plus opened loop sensor or hypo minimiser. HCL: Hybrid closed loop pumps. The blue and red lines are coefficients and 95% CI of clusters hyperglycaemia and hypoglycaemia, respectively. Cluster euglycaemia is the reference.

ESM Fig. 7. Bayesian network plot

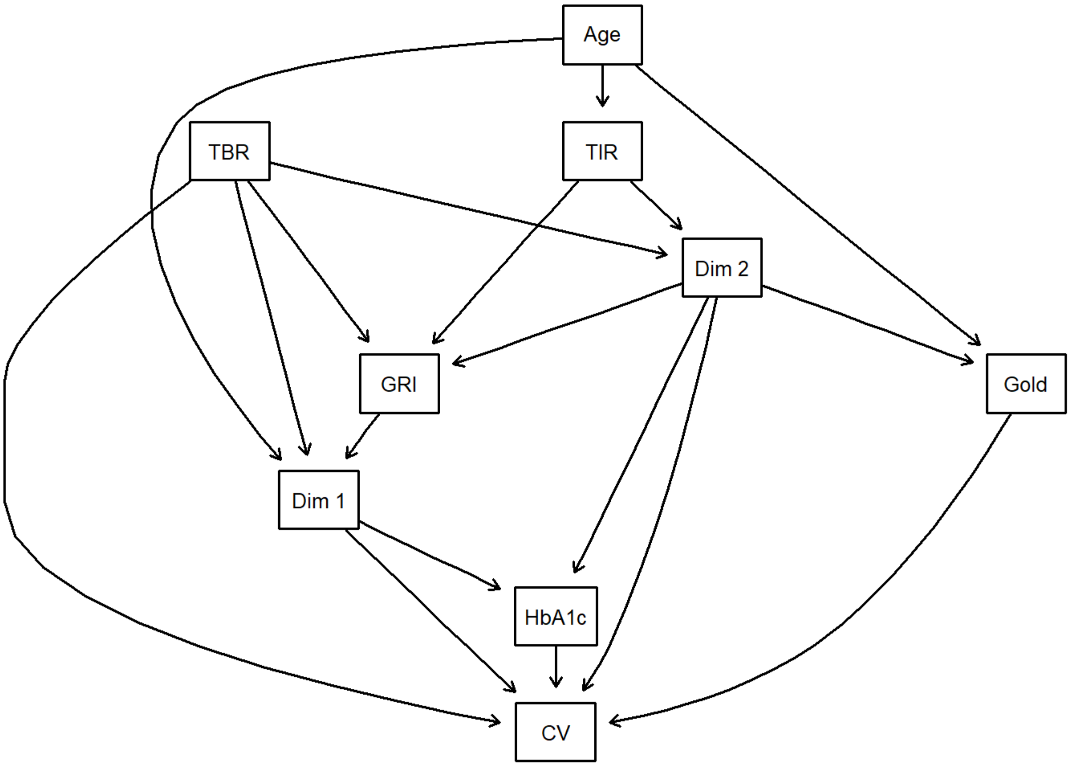

The arrows represent directed associations among the features.

Abbreviations: Dim1: Dimension 1; Dim2: Dimension 2; TIR: time in range; TBR: time below range; CV: coefficient of variation; GRI: Glycaemic risk index; Gold: Gold score.

ESM Fig. 8. Network plot

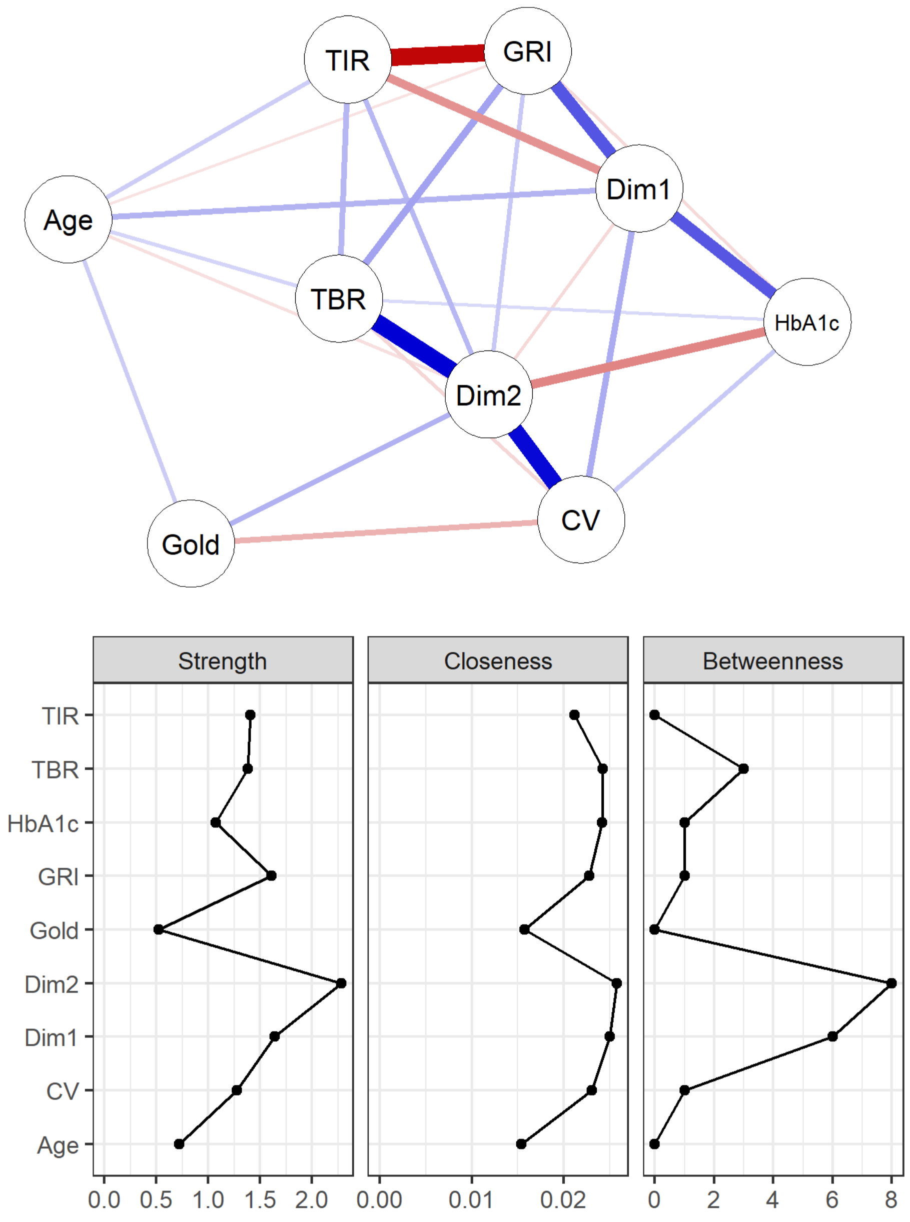

Superior panel shows the feature network. Blue lines represent direct associations and orange line inverse associations. The width of the edges relates to the magnitude of association between them (e.g. partial correlation). Panel below shows measures of centrality, i.e. the relative importance of each node to the whole network: strength, closeness and betweenness. Dim2 shows the highest centrality in the network.

Abbreviations: Dim1: Dimension 1; Dim2: Dimension 2; TIR: time in range; TBR: time below range; CV: coefficient of variation; GRI: Glycaemic risk index; Gold: Gold score.
